# Functionalized Cellulose for the Controlled Synthesis of Novel Carbon–Ti Nanocomposites: Physicochemical and Photocatalytic Properties

**DOI:** 10.3390/nano10040729

**Published:** 2020-04-11

**Authors:** Hesham Hamad, Esther Bailón-García, Sergio Morales-Torres, Francisco Carrasco-Marín, Agustín F. Pérez-Cadenas, Francisco J. Maldonado-Hódar

**Affiliations:** 1 Carbon Materials Research Group, Department of Inorganic Chemistry, Faculty of Sciences, University of Granada, Avda, Fuente Nueva, s/n. ES18071 Granada, Spain; heshamaterials@hotmail.com (H.H.); estherbg@ugr.es (E.B.-G.); fmarin@ugr.es (F.C.-M.); afperez@ugr.es (A.F.P.-C.); fjmaldon@ugr.es (F.J.M.-H.); 2 Fabrication Technology Department, Advanced Technology and New Materials Research Institute (ATNMRI), City of Scientific Research and Technology Applications (SRTA-City), New Borg El-Arab City 21934, Egypt

**Keywords:** cellulose decrystallization, phosphorus functionalities, carbon–Ti nanocomposites, TiP_2_O_7_ crystals, Orange G, photocatalysis

## Abstract

Carbon–Ti nanocomposites were prepared by a controlled two-step method using microcrystalline cellulose as a raw material. The synthesis procedure involves the solubilization of cellulose by an acid treatment (H_3_PO_4_ or HNO_3_) and the impregnation with the Ti precursor followed of a carbonization step at 500 or 800 °C. The type of acid treatment leads to a different functionalization of cellulose with phosphorus- or oxygen-containing surface groups, which are able to control the load, dispersion and crystalline phase of Ti during the composite preparation. Thus, phosphorus functionalities lead to amorphous carbon–Ti composites at 500 °C, while TiP_2_O_7_ crystals are formed when prepared at 800 °C. On the contrary, oxygenated groups induce the formation of TiO_2_ rutile at an unusually low temperature (500 °C), while an increase of carbonization temperature promotes a progressive crystal growth. The removal of Orange G (OG) azo dye in aqueous solution, as target pollutant, was used to determine the adsorptive and photocatalytic efficiencies, with all composites being more active than the benchmark TiO_2_ material (Degussa P25). Carbon–Ti nanocomposites with a developed micro-mesoporosity, reduced band gap and TiO_2_ rutile phase were the most active in the photodegradation of OG under ultraviolet irradiation.

## 1. Introduction

The occurrence of organic contaminants at very low levels in different water sources, including drinking water, has emerged as a global concern. Azo dyes represent about 70% of the synthetic chemical dyes and possess a relatively stable chemical structure [1], composed by aromatic rings, which confer them with carcinogenic and mutagenic properties [2]. Thus, effluents containing this contaminant need to be treated by advanced oxidation processes (AOPs), because conventional treatments used in municipal wastewater treatment plants (MWWTPs) are not completely efficient for their removal. Heterogeneous photocatalysis has been demonstrated to be effective in the removal of pollutants from air [3] and water [4] in recent years. Most of studies are based on titanium dioxide (TiO_2_) because of its relatively low price and toxicity and its high stability and recyclability [5,6]. However, its practical application is severely compromised by the low quantum yield and the poor light-harvesting ability as consequence of its wide band gap (i.e., 3.2 or 3.0 eV for anatase or rutile phases, respectively) [7].

Different alternatives have been proposed to modify the band gap of TiO_2_, either with metals (Al, Gd, Zr, W, Ag) [8,9] or nonmetallic (S, N, P) dopants [10,11]. The recombination of photo-generated charges can be enhanced by reduction of the TiO_2_ particle size through the synthesis procedure [12]. One of the most interesting strategies is the development of TiO_2_ composites with carbon materials, due to favoring a better dispersion of TiO_2_ nanoparticles, reducing the TiO_2_ particle size, acting as photosensitizers and enhancing the light absorption and pollutant adsorption [13,14]. In this way, different types or carbon materials, from classical activated carbons [15], to nanocarbons, including xerogels, nanotubes, fullerenes or graphene derivatives, have been employed to develop efficient photocatalysts due to their excellent electronic, optical and porous properties [4,16,17].

On the other hand, suitable synthetic methods and cheap, recyclable and environmentally friendly precursors, like biopolymers, should be considered for the development of TiO_2_ composites [18,19,20]. In this context, cellulose is the cheapest and the most abundant renewable material obtained directly from biomass. However, most of studies deal with the immobilization of TiO_2_ particles on cellulose substrates, such as fabrics or fibers [21,22], and a few works were published about cellulose–TiO_2_ composites for water treatment [23,24,25,26,27]. Yet, to the best of our knowledge, the role of chemical functionalities of cellulose on the properties and photocatalytic performance of carbon–Ti composites has not been explored.

The aim of this work is to develop carbon–Ti composites looking for the synergism between phases using cheap, abundant and sustainable microcrystalline cellulose (MCC) raw material to increase the photo-response and adsorption capacity. The synthesis procedure, in particular the acid pretreatment of cellulose, the subsequent heteroatom-doping and the physicochemical transformations during carbonization, was optimized for the sake of improving the carbon–Ti interactions. Carbon–Ti composites were obtained by a two-step method using MCC as a carbon precursor and structure-directing agent. The synthesis procedure involves the previous MCC solution by an acid treatment with H_3_PO_4_ or HNO_3_ and the impregnation with the Ti precursor followed by a thermal treatment. Results showed that during the acid treatment, the cellulose chains are functionalized with phosphorus- or oxygen-containing surface groups, which are able to control the Ti loading and dispersion during impregnation and stabilize different Ti phases and structures during carbonization. The adsorptive and photocatalytic behaviors of the materials for the removal of Orange G (OG) in aqueous solution were studied and correlated with the chemical, physical and crystalline transformations achieved for each composite depending on the acid pretreatment and carbonization temperature.

## 2. Materials and Methods

### 2.1. Chemicals

Microcrystalline cellulose (MCC) and titanium (IV) isopropoxide (TTIP) were purchased from Merck (Darmstadt, Germany), while o-phosphoric acid (85% w/w), nitric acid (70% w/w) and heptane were acquired from VWR chemicals (Leuven, Belgium). Orange G (OG, C_16_H_10_N_2_Na_2_O_7_S_2_), also known as Acid Orange 10, was obtained from Acros Organics (Geel, Belgium).

### 2.2. Synthesis of Carbon–Ti Composites

An aqueous suspension of MCC (200 g·L^−1^) was prepared and stirred for 15 min at 50 °C, and then 10 mL of concentrated acid, i.e., 15.6 M HNO_3_ or 14.8 M H_3_PO_4_, was added dropwise and left overnight. The Ti impregnation of the MCC solution was carried out by dropping a heptane solution of TTIP (cellulose–TiO_2_ mass ratio = 1:6). The suspension formed was aged at 60 °C for 24 h, and then the solid was filtered, washed with DI water and acetone, dried at 120 °C in an oven and finally carbonized in a tubular furnace at 500 or 800 °C in N_2_ flow (100 cm^3^·min^−1^). The carbon–Ti composites were labeled as CXTiY, where “X” corresponds to the acid used, i.e., “P” or “N” for H_3_PO_4_ or HNO_3_, respectively, “Ti” indicates the presence of Ti phase and “Y” the carbonization temperature, i.e., “1” or “2” for 500 or 800 °C, respectively. For instance, the CNTi2 sample is a carbon–Ti composite prepared with cellulose treated with HNO_3_ and carbonized at 800 °C. Additionally, pure carbon materials, i.e., CN1, CN2, CP1 and CP2 samples, were also prepared as reference following a similar procedure but not adding any amount of Ti precursor.

### 2.3. Characterization Techniques

The morphology of materials was analyzed by scanning (AURIGA (FIB-FESEM) microscope, Carl Zeiss SMT, Oberkochen, Germany) and transmission electron microscope (HAADF FEI TITAN G2, Oberkochen, Germany). Textural characterization was carried out by physical adsorption of N_2_ at −196 °C using a Quantachrome Autosorb-1 equipment. The BET method and the Dubinin–Radushkevich equation were applied to determine the apparent surface area (*S_BET_*) and the micropore volume (*W_0_*), respectively. The microporous surface (*S_micro_*) and the mean micropore width (*L_0_*) were estimated from the Stoeckli equation [28,29], while the mesopore volume (*V_meso_*) was calculated by applying the BJH method [30]. Prior to the N_2_ adsorption measurements, the samples were outgassed overnight under a dynamic vacuum of 10^−6^ mbar at 120 °C.

The carbonization step and TiO_2_ content of composites was determined by thermogravimetric (TG) analysis under N_2_ or air flow at 5 °C·min^−1^ using a Mettler-Toledo TGA/DSC1 thermal balance (Mettler-Toledo International Inc., Greifensee, Switzerland). The crystallographic phase of materials was determined by X-ray diffraction (XRD) using a Bruker D8 Advance X-ray diffractometer (Cu Kα radiation, λ = 1.541 Å, BRUKER, Rivas-Vaciamadrid, Spain). X-ray photoelectron spectroscopy (XPS) spectra of C1s, O1s, N1s, P2p and Ti2p regions were obtained on a Kratos Axis Ultra-DLD X-ray photoelectron spectrometer (Kratos Analytical Ltd., Kyoto, Japan), and ultraviolet–visible (UV/Vis) diffuse reflectance spectra (DRUV) were obtained using a double-beam UV/Vis spectrophotometer (CARY 5E from VARIAN, Markham, Canada) [31]. The pH_PZC_ values were determined following a previously published methodology [17] and correspond to the equilibrium pH for 100 mg of the material dispersed in 5 mL of water (at 25 °C).

### 2.4. Photocatalytic Tests

The adsorption and photocatalytic behaviours of carbon–Ti composites were evaluated for the degradation of 10 mg·L^−1^ (2.21 × 10^−5^ mol·L^−1^) OG at room temperature under UV irradiation. The batch experiments were carried out using a glass cylindrical reactor with 800 mL of OG solution, using a load catalyst of 1.0 g·L^−1^ and a low-pressure mercury vapor lamp (TNN 15/32, 15 W, λ = 254 nm, HNG-Germany). Samples at different reaction times were withdrawn and centrifugated to separate the catalyst particles before analysis. Prior to photocatalytic tests, all materials were saturated with the OG solution in dark to remove the adsorptive contribution from the evolution of the total OG concentration. After dark phase, the initial OG concentration (*C_0_*) was adjusted again to 10 mg·L^−1^, and then a UV lamp was turned on, this time being considered t = 0. The concentration of OG was monitored by a UV/Vis spectrophotometer (5625 Unicam Ltd., Cambridge, UK). 

## 3. Results and Discussion

### 3.1. Materials Characterization

**Figure 1:**
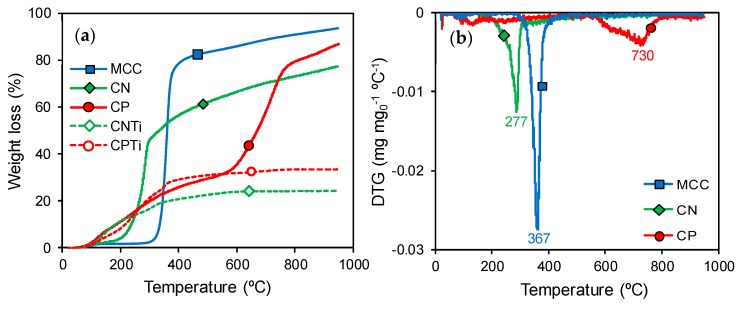
(**a**) Thermogravimetric (TG) profiles under N_2_ flow for microcrystalline cellulose (MCC) and acid-treated cellulose samples before (solid lines) and after Ti impregnation (dash lines); (**b**) Differential thermogravimetric (DTG) profiles under N_2_ flow for MCC, CN and CP.

Carbon–Ti composites were prepared after carbonization of cellulose samples impregnated with the Ti precursor. A MCC solution was first prepared by an acid treatment (i.e., HNO_3_ or H_3_PO_4_) to facilitate the impregnation and dispersion of the Ti phase. The carbonization processes of raw MCC (commercial) and acid-treated celluloses (CN and CP) were simulated by TG analysis under N_2_ flow and used as reference processes, pointing out the strong changes on the thermal stability of cellulose after HNO_3_ or H_3_PO_4_ pretreatments. The thermal decomposition of the raw cellulose (MCC) occurs in a single step at around 340 °C (Figure 1a), in agreement with that previously reported [32] and indicating the absence of both hemicellulose and lignin. The theoretical total carbon content is 44 wt. %, taking into account the chemical composition of cellulose (C_6_H_10_O_5_)_n_. However, the yield of carbon residue after pyrolysis is significantly lower (around 17 wt. %) because the depolymerization of the cellulose molecular structure takes place during heating and evolves carbon oxides (CO and CO_2_) from intermediate aldehydes and carboxylic acids, consequently decreasing the yield of carbon residues [33].

The thermal decomposition of acid-treated cellulose presents significant differences regarding the original MCC. After the HNO_3_ treatment, the CN sample decomposes mainly in one step, like MCC, but at a lower temperature (Figure 1a). This lower thermal stability is associated with the incorporation of new oxygen chemical functionalities, in particular carboxylic acid groups. Dehydration processes of adjacent carboxylic acid groups and/or the decomposition of these groups into CO_2_ occurs at temperatures below 300 °C [34]. Nevertheless, after this main decomposition step, a slow and continuous weight loss also occurs with increasing temperature, indicating the decomposition of additional oxygenated surface groups that typically evolve as CO (phenol, carbonyl or quinone) [34]. After the H_3_PO_4_ treatment, carbonization takes place clearly in two steps placed at around 200 and 700 °C. The first decomposition step may be also due to dehydration and decomposition of derivative groups of carboxylic acids and anhydrides, but the decomposition temperature range (from around 150 to 500 °C) is narrower in CP than in MCC or CN samples, indicating a more heterogeneous distribution of surface groups. The second carbonization step is associated with the reduction of phosphate groups by the organic cellulose, which increases the cellulose gasification in this temperature range. Chemical activation of H_3_PO_4_-impregnated lignocellulosic materials [35,36] is a classical method for the preparation of activated carbons, with the activation temperature depending on the nature of the raw biopolymer [37]. In the case of the CP sample, the weight loss associated with the activation process occurs at 700 °C, while, as an example, coconut activation has been reported between 400–450 °C [35]

Acid-treated cellulose samples showed an obvious yield of solid residue that was markedly greater after their impregnation with the Ti precursor (Figure 1a). The significant change in the TG profiles of carbon–Ti composites (CNTi and CPTi) regarding their corresponding supports (CN and CP) is noteworthy, denoting the formation of new bonds between the cellulose supports and the Ti precursor. At low temperature (below 200 °C), the weight loss in the CNTi sample even increases regarding CN by decomposition of organic precursors, but after that the slope of the curve strongly decreases; there is not a significant weight loss from around 600 °C, showing the thermal stabilization of the cellulose oxygenated surface groups by interaction with the Ti phases. The cellulose–Ti interactions are also observed for the CPTi sample. In this case, the first decomposition step remains more or less unchanged, but the second decomposition step is not observed, therefore indicating that the phosphate groups are not reduced by the organic cellulose phase during the thermal treatments and thus are also clearly stabilized by reactions with the Ti phase.

The inorganic content in the composites was also determined by TG, in this case by burning a small portion of each sample in air flow. Although both samples were prepared using a constant cellulose:TiO_2_ ratio of 1:6 by weight, the residue percentage was 63 and 74 wt. % for CNTi and CPTi, respectively. Thus, the highest residue content determined for CPTi is favored by the presence of phosphorus functionalities. The surface chemical nature and composition of Ti-impregnated cellulose and its carbon derivatives were analyzed by measurements of pH_PZC_ and XPS (Table 1). The influence of acid treatments on pH_PZC_ of the carbon–Ti composites is noteworthy, as CPTi samples are strongly acidic materials, while the surface chemistry for CNTi samples is close to neutrality. The highest oxygen content detected on the surface of the CPTi sample regarding the CNTi sample, together with the high phosphorus content, suggests the presence of phosphate groups. However, the Ti content of CPTi is lower than in CNTi despite the highest solid residue detected by TG, therefore indicating a different Ti distribution in the surface than in the sample bulk. During carbonization, CPTi composites undergo stronger changes than CNTi samples, increasing the carbon content and decreasing the oxygen and Ti contents (Table 1). The low N content detected in CNTi (Table 1) was removed after carbonization (CNTi2) and should not influence its catalytic performance.

**Figure 2:**
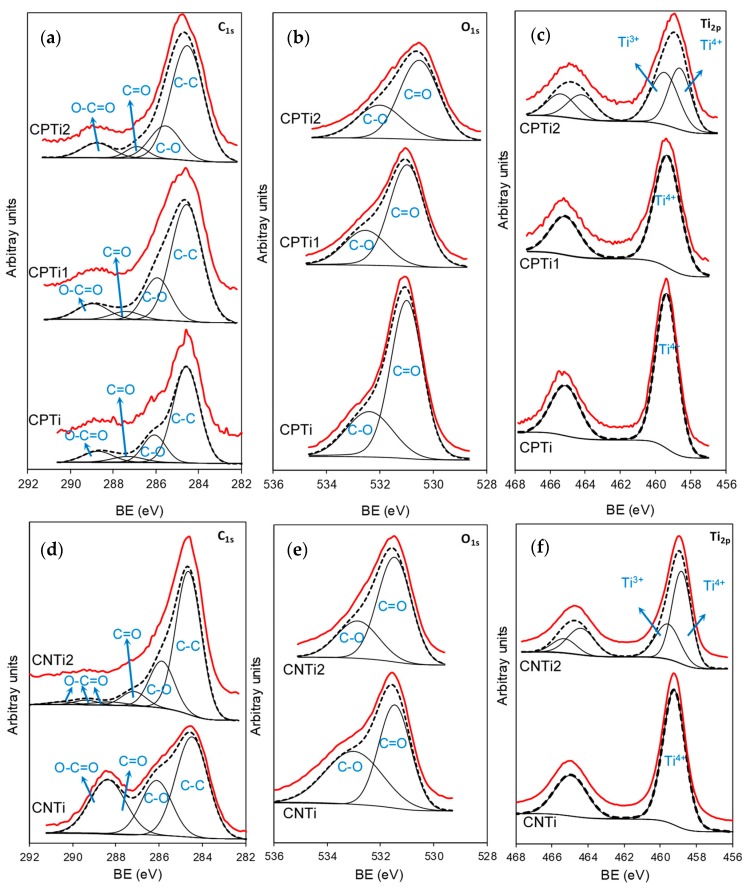
High-resolution XPS spectra of the C1s (**a**,**d**), O1s (**b**,**e**) and Ti2p (**c**,**f**) regions for Ti-impregnated cellulose derivatives and their corresponding carbonized composites.

The XPS results also show the interactions between the chemical groups of cellulose and the Ti phase, as well the transformations performed during carbonization. The XPS spectra of C1s, O1s and Ti2p regions for the Ti-impregnated samples before (CPTi and CNTi) and after carbonization at different temperatures (CPTi1, CPTi2 and CNTi2) are collected in Figure 2. In general, the deconvolution of the C1s region shows a main contribution at 284.6 eV associated to C–C bonds and other smaller peaks at higher binding energies (BE) due to the occurrence of oxygenated surface groups, i.e., C–O, C=O or carboxyl groups (Figure 2a,d) [36,38]. These small components are more significative in cellulose treated with HNO_3_ (CNTi) than in CPTi. After carbonization, the peaks due to oxygen-containing groups are maintained in CPTi1 and CPTi2 samples, while a strong decrease is observed for CNTi2 because of the weak thermal stability of carboxylic acid groups introduced by HNO_3_ compared to the phosphate groups detected for CPTi samples.

The O1s spectrum was deconvoluted in two components located at ~531.5 and ~533.1 eV, corresponding to double-bonded oxygen (e.g., carbonyl and carboxyl groups) and single-bonded oxygen (e.g., alcohol groups), respectively (Figure 2b,e) [39]. Moreover, Ti–O and Ti–OH bonds should contribute to both first and second peaks, respectively [40]. All these contributions also decrease after carbonization, mainly for the case of CNTi2, as observed for the C1s spectrum. The analysis of the P2p spectrum also allows to point out the interaction of Ti phases with the phosphorus-containing groups incorporated during the cellulose treatment. The P2p region can be deconvoluted in only a peak at ~133.4 eV for CPTi, while a second peak is found at ~134.7 eV after carbonization (Figure S1 of the Supplementary Materials). In general, the peaks placed at ca. 134 eV and 135 eV are usually due to phosphorus linked to carbon (C–PO_3_) and to pentavalent tetracoordinated phosphorus in phosphates or polyphosphates (C–O–PO_3_), respectively [41]. Therefore, H_3_PO_4_ treatments incorporate oxygen- and phosphorus-containing functionalities, while only oxygenated groups are detected for samples treated by HNO_3_.

Regarding the Ti2p region (Figure 2c,f), only a peak at 459.4 eV corresponding to Ti^4+^ is detected on the Ti-impregnated samples (CNTi and CPTi) and on the carbonized composites at low temperature (e.g., CPTi1). However, the spectra of both CNTi2 and CPTi2 are wider and shifted to lower BE values, showing the presence of a mixture of Ti^4+^ and Ti^3+^ and denoting the influence of the carbonization temperature on the nature of the Ti phase. Despite the certain reduction observed, the difference in BE between Ti 2p_3/2_ and Ti 2p_1/2_ components is always maintained at 5.7 eV, in agreement with TiO_2_ results previously reported [42].

**Figure 3:**
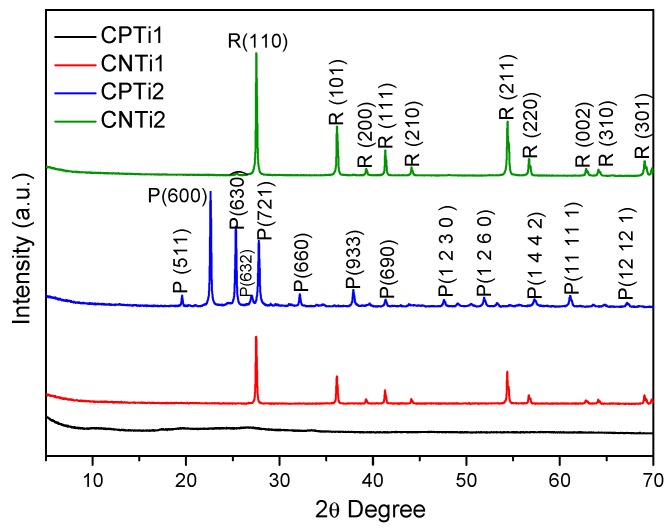
X-ray diffraction (XRD) patterns of the carbon–Ti composites (R = rutile, P = TiP_2_O_7_).

The analysis of the XRD patterns also pointed out the different transformations of the samples as a function of the carbonization temperature in the composites (Figure 3) and, consequently, the different metal–support interactions. Thus, the absence of XRD peaks is observed for CPTi carbonized at 500 °C (CPTi1), while XRD patterns denoting a total transformation to the rutile phase can be detected in the case of CNTi1. In previous works [43,44], the carbon phase was reported to prevent both the sintering and phase transformation of the inorganic oxides in carbon/oxide composites, including TiO_2_, SiO_2_ or Al_2_O_3_/C. In this case, the phosphate groups on the cellulose support should avoid the crystal growth during carbonization at low temperatures. However, the formation of rutile at 500 °C observed for CNTi1 was not expected, taking into account that the transformation of the anatase phase to rutile normally occurs at 750 °C, although it can be induced at lower temperatures in some cases, such as by doping with metals like V^5+^ [45]. By increasing the carbonization temperature up to 800 °C, an increase of the rutile crystallinity is observed in CNTi2 compared to CNT1, as denoted by the narrower and more intense diffraction peaks. Therefore, XRD patterns for CNTi1 and CNTi2 samples clearly showed the presence of only rutile (JCPDS Card No. 21-1276) peaks placed at 2*θ* values of ~27°, ~36°, ~41°, ~44°, ~54°, ~56° and ~61°, which correspond to crystal planes of (110), (101), (200), (111), (210), (211), (220), (002) and (310) [46]. At this carbonization temperature, the CPTi2 XRD pattern showed the formation of a crystalline phase that does not correspond to any TiO_2_ phase, but it was indexed as cubic TiP_2_O_7_ with the space group Pa3 and 3 × 3 × 3 superstructure (JCPDS No. 38-1468) [47,48]. The formation of TiP_2_O_7_ nanocrystals is only obtained at temperatures higher than 1000 °C [49]; thereby, it should be favored by the phosphorus surface groups on the functionalized cellulose. These results are also in agreement with the TG and XPS results, which suggested the stabilization of the phosphate groups by interaction (reaction) with the Ti species with increasing temperature. In summary, the functionalities incorporated during cellulose solubilization determine the Ti phase formed in the corresponding composites. The oxygenated surface groups, namely carboxylic acid species, generated on the cellulose chains by HNO_3_ treatments induce the formation of rutile at low temperature, while the phosphorus groups generated by H_3_PO_4_ treatments avoid the formation of crystalline Ti phases when treated at low temperatures and the formation of TiP_2_O_7_ nanocrystals with increasing this parameter, but the reaction occurs at lower temperatures than those typically reported.

**Figure 4:**
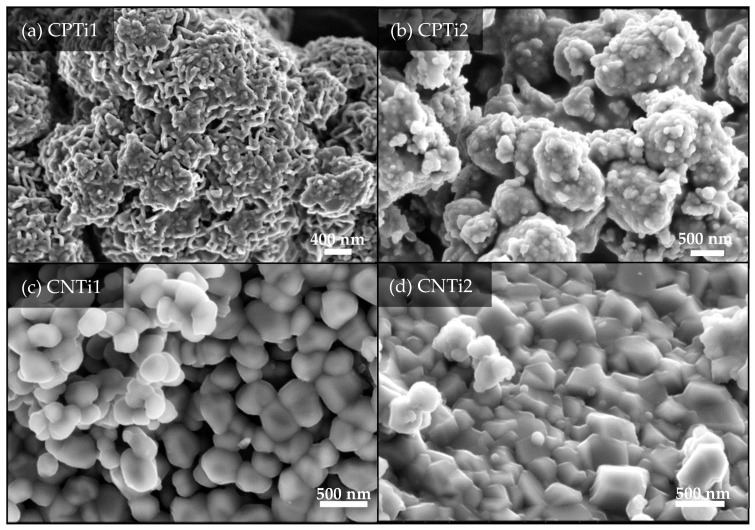
Scanning electron microscopy (SEM) images of the carbon–Ti composites prepared by cellulose treatment with phosphoric acid (**a**,**b**), or nitric acid (**c**,**d**).

The chemical and crystalline transformations previously indicated are simultaneously accompanied by a marked change in the morphology of the samples, mainly for the CPTi carbonized derivatives. Thus, the CPTi sample carbonized at low temperature (500 °C) presents an opened structure composed of grouping sheets leading to a solid with flaky aspect (Figure 4a). When the composite is carbonized at 800 °C (CPTi2, Figure 4b), the structure becomes more compact, showing a granular aspect that corresponds to the formation of the TiP_2_O_7_ crystalline phase, according to XRD results. The morphology of CNTi1 is formed by round-shaped particles overlapping and forming an opened structure (Figure 4c). These particles should correspond to rutile crystals identified by XRD (Figure 3). The composite carbonized at 800 °C (CNTi2) also presents a denser surface than CNTi1 by sintering, and the crystallites now show sharp edges on clearly cubic structures due to the mentioned crystal growth (Figure 4d).

**Figure 5:**
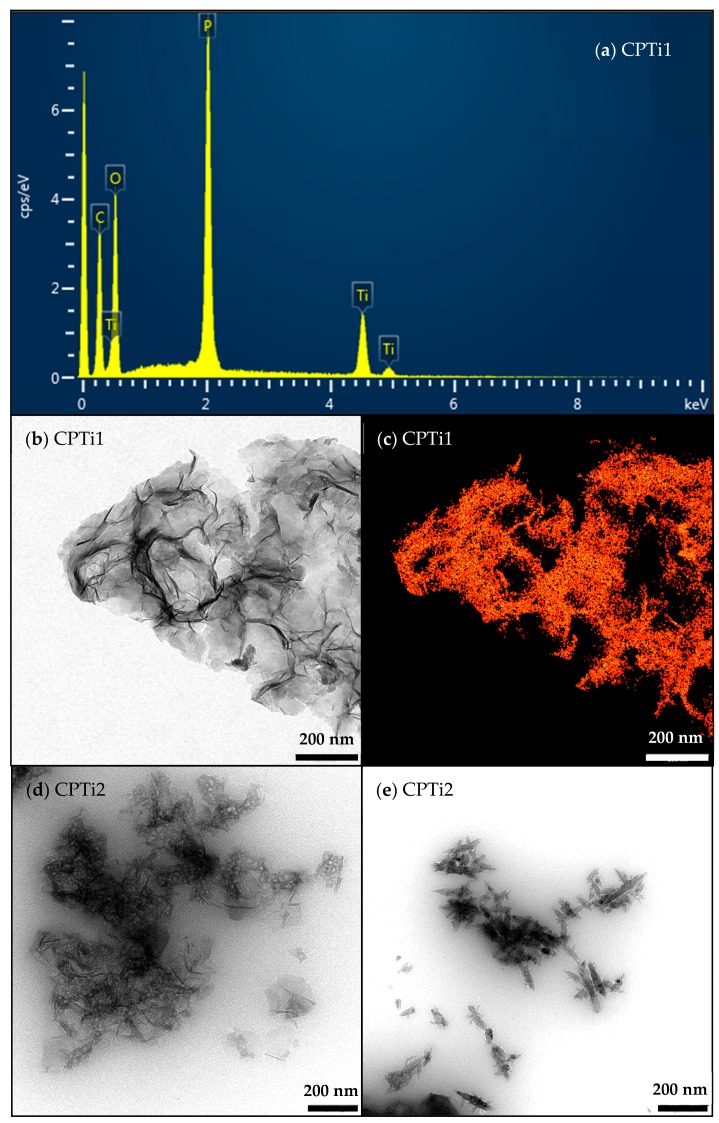
High resolution transmission electron microscopy (HRTEM) characterization of CPTi composites: (**a**) energy dispersive X-ray (EDX) analysis, (**b**,**d**,**e**) micrographs and (**c**) Ti-mapping.

The energy dispersive X-ray (EDX) spectrum corroborates the presence of C–O–P–Ti in the CPTi composite (Figure 5a). High resolution transmission electron microscopy (HRTEM) micrographs of CPTi1 (Figure 5b) shows the formation of ultrathin carbon layers, in which a homogeneous distribution of Ti is deposited throughout the composite, as confirmed by mapping (Figure 5c). After carbonization at 800 °C, the formation of needle-shaped structures, more or less aggregated, are also observed, together with the large carbon flakes previously described (Figure 5d,e). These flat and transparent carbon structures were also previously observed after sulfuric acid hydrolysis and carbonization of *Eucalyptus* kraft pulp [50]. Filaments, aggregates of films, can be developed depending on the thermal treatments of cellulose nanocrystals, although the mechanism of formation of these nanostructures is still unclear. It seems to be related with the H-bonding interactions in each media and the transformations during carbonization.

**Figure 6:**
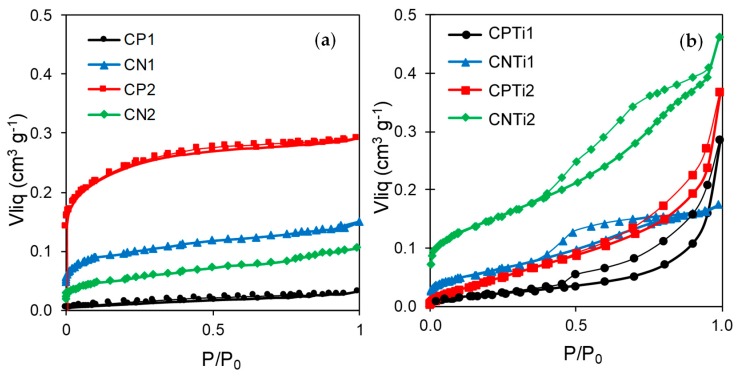
N_2_ adsorption–desorption isotherms of (**a**) the pure carbon materials and (**b**) the carbon–Ti composites.

The different morphologies observed for the carbon–Ti composites influence their porosity, which was analyzed by N_2_ adsorption–desorption isotherms (Figure 6). The shape of the isotherms already points out the different porosity of the samples. When comparing the isotherms of pure carbon samples (Figure 6a) and their corresponding composites (Figure 6b), a different porous texture due to the interactions and transformations between phases is clearly visible. Thus, the porosity of the CP sample carbonized at 500 °C (i.e., CP1) is quite poor (i.e., *S_BET_* = 17 m^2^·g^−1^), while CP2, carbonized at 800 °C, presents a high surface area (i.e., *S_BET_* = 552 m^2^·g^−1^), associated to the microporosity developed during the activation processes by the reduction of the phosphate groups at ~700 °C, as previously observed by TG. In fact, CP2 is mainly microporous, as denoted by its type-I adsorption isotherm (Figure 6a). On contrary, the CN1 sample, pretreated in nitric acid and carbonized at low temperature, showed a higher porosity than CP1 (i.e., *S_BET_* = 226 vs. 17 m^2^·g^−1^, respectively), due to the evolution of low-stability oxygenated surface groups. However, the porosity and surface area of CN samples decrease after increasing carbonization temperature with a progressive micropore widening (*L_0_*, Table 1). CN1 and CN2 samples showed type-II adsorption isotherms.

After Ti precursor impregnation, both CNTi1 and CNTi2 showed type-IV adsorption isotherms characteristic of micro-mesoporous materials, while mainly type-II isotherms were obtained for both CPTi1 and CPTi2. These samples showed a similar microporosity (*W_0_*, Table 2) and surface area (*S_micro_*, Table 2), in contrast to the previous nonimpregnated samples (e.g., CP1 and CP2), which corroborates that the interaction of the carbon with the Ti phase avoids the reduction of the phosphate groups. Nevertheless, a certain delay in the desorption branch of both CPTi1 and CPTi2 samples also denotes a hysteresis cycle associated to N_2_ condensation. In all composites, the shape of the isotherm is maintained while increasing the carbonization temperature_,_ but the total porosity and BET surface areas increase (Table 2). These increases are due to a larger weight loss and, on the other hand, to the progressive sintering and crystal growth of the Ti phase (Figure 4 and Figure 5) leading to narrow pores where N_2_ condensates during the porosity filling. CNTi derivative samples mainly present a developed micropore structure that strongly favors high surface area values compared to those for the CPTi ones. In addition, CNTi2 presented the largest development of porosity, with sintering favoring the formation of mesopores (*V_meso_*, Table 2) from macropores or interparticle voids present in the sample obtained at lower carbonization temperature. Porosity, namely mesoporosity (*V_meso_*), is favored in the carbon–Ti composites in regard to their pure carbon materials, an evolution directly related to the transformations of the Ti phase being established.

**Figure 7:**
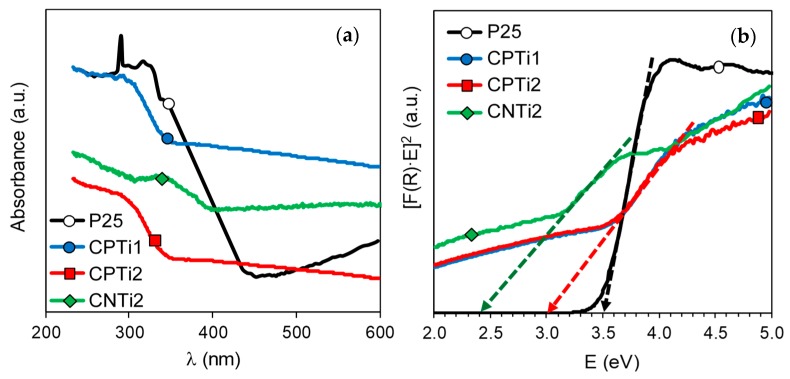
(**a**) UV/Vis diffuse reflectance spectra (DRUV) of P25 and different carbon–Ti composites. (**b**) Plot of transformed Kubelka–Munk units as a function of the energy of light (inset: determination of the band gap).

The optical properties of the carbon–Ti composites were analyzed using UV/Vis diffuse reflectance (DRUV) spectroscopy. All reflectance spectra (Figure 7a) recorded were converted to equivalent absorption Kubelka–Munk (K-M) units (Figure 7b) and the band gap (Eg) was determined from the plots of (F(R) × h*ν*)^n^ vs. E by extrapolating the slope to a = 0 according to the procedure previously reported [4,51,52]. The band gap for all composites synthesized from cellulose decreased in regard to the value determined for P25, which was used as reference material (Figure 7b). CNTi samples, with rutile as active phase, presented Eg values at ~2.4 eV regardless of the carbonization temperature; this value of band gap was significantly lower than 3.0 eV, corresponding to the pure rutile phase. Analogously, the strong influence of the carbonization temperature on the crystalline and morphological properties previously discussed for CPTi composites does not produce a significant change in the profile of their corresponding spectra; consequently, similar band gap values, at ~3.0 eV, are obtained (Figure 7b) for CPTi1 and CPTi2. However, these values are also significantly smaller than the band gap of 3.5 eV reported in the bibliography for the TiP_2_O_7_ phase [52]. Thus, a clear influence of the functionalized cellulose on the semiconductor properties of both series of composites is evidenced. On the other hand, the carbonization temperature did not markedly influence the band gap values of the carbon–Ti composites, despite presenting different physicochemical properties. Different effects can contribute to the similar band gap values observed. For instance, with increasing carbonization temperature, the formation of Ti^3+^ species were pointed out by XPS, which can contribute to the bad gap narrowing. However, this also progressively increases the crystal size of the composites, which evidently favors the electron–hole recombination. Otherwise, the low band gaps determined for all composites should significantly improve their photocatalytic efficiency compared to the pure TiO_2_ materials, such as P25.

### 3.2. Removal of Orange G (OG) Azo Dye

**Figure 8:**
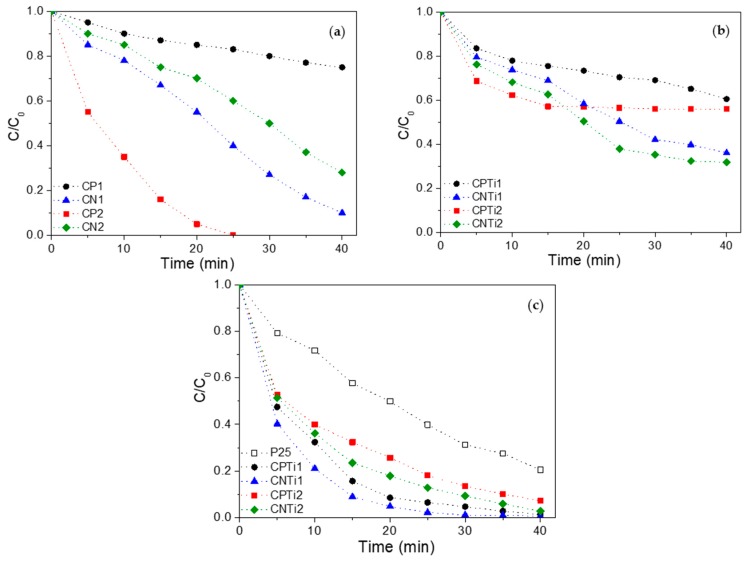
Removal of Orange G (OG) from water solution by (**a**) adsorption using pure carbon materials and (**b**) adsorption and (**c**) photocatalysis using carbon–Ti composites.

The adsorptive and photocatalytic performance of the carbon–Ti composites was analyzed for the removal of OG, a textile dye typically present in industrial wastewater and used as target molecule, following the procedure described in our previous works with other semiconductors [4,53]. Firstly, all samples were saturated for 4 h using a concentrated OG solution in dark experiments, which allows to analyze the interactions of pollutants with the sample surface but also avoids the contribution of the adsorption process on the OG removal during the subsequent photocatalytic degradation experiment. The initial adsorption kinetic curves for pure carbon materials and the corresponding composites are shown in Figure 8a,b, respectively.

The analysis of OG-adsorptive performance of the pure carbon materials allowed a direct relationship between the OG removal and the micropore volume (*W_0_*, Table 2) of the samples to be established. The high micropore development in CP2 allows the complete removal of OG in solution in only 25 min, while CP1 (with the lowest W_0_) leads to the worst adsorptive behavior (Figure 8a). In the case of CN materials, the OG-adsorption capacity decreases as carbonization temperature increases due to a micropore destruction, probably by widening (Table 2). For the case of composites, CPTi2 also showed a better performance than CPTi1, although the microporosity of both samples is quite similar (Figure 8b). The increased microporosity with the carbonization temperature explains the better OG-adsorptive performance of CNTi2 compared to CNTi1 (Figure 8b). In these composites, the mesoporosity also increased with the carbonization temperature, which can favor the accessibility and adsorptive performance of the samples. The fast adsorption processes of the CPTi2 composite could be favored by chemical interactions of the carbon surface with the dye in solution. The beneficial effect on the dye adsorption of phosphate doping in Ti structures was previously reported [29,54] as due to the modification of the electrostatic interactions. Nevertheless, the best final performance of CNTi samples confirms the importance of a developed porosity in the interactions with the pollutant.

Once the carbon–Ti composites were saturated with the OG solution, the photocatalytic performance of the composites was studied (Figure 8c), with the best efficiency being observed for the CNTi1 sample. In the CNTi composite series, the catalytic activity decreases with increasing carbonization temperature, despite the higher porosity and similar band gap values of CNTi2. A similar influence of the carbonization temperature on the catalytic activity can be described for CPTi1 and CPTi2 composites. However, all carbon–Ti composites present a better performance than the reference TiO_2_ photocatalyst (P25), reaching total OG removal in 40–60 min. The activity order follows the sequence CNTi > CPTi > P25, in agreement with the evolution of the band gap values. This fact should be associated with the increasing crystallinity of the rutile or TiP_2_O_7_ phases and confirms the importance of the particle size in the interaction with the support and the electron–hole recombination processes. The reaction pathway for the photodegradation of the azo-dye acid Orange 7 in TiO_2_ suspensions was studied by Stylidi et al. [55], reporting that the mineralization to CO_2_ occurs with the formation of aromatic and aliphatic acids as intermediates. In previous works [4,53], we have pointed out that the photocatalytic experiments carried out using C–Ti or C–Zr composites produce the mineralization of the dye to CO_2_. In the current work, a similar behavior was observed; no intermediates were expected in solution after reaction, because the TOC analysis of the samples decreased in accordance with the OG removal. However, the study of the reaction mechanism should be better investigated in future works considering the stability of the most active photocatalysts during consecutive reaction cycles. The best photocatalytic performance of the CNTi1 sample seems to be related to an adequate porous texture, the narrow band gap associated to the rutile phase in combination with the carbon support and an intermediate particle size. In the case of the CPTi1 composite, the low surface area can be compensated by the favorable role of phosphate groups on the adsorption and a very small particle size (not detectable by XRD). Therefore, this study demonstrates the possibility to enhance the photocatalytic activity of TiO_2_ by preparing composites from biopolymers, like cellulose, through an easy procedure, highlighting the importance of the cellulose pretreatment conditions on the final properties and catalytic performance. Even so, the developed photocatalysts presented a good activity compared to other materials reported in literature (Table 3).

## 4. Conclusions

Cheap and sustainable carbon–Ti composites with improved adsorptive and photocatalytic performances were obtained using cellulose as raw material. The oxygen- and phosphorus-containing surface groups generated on the cellulose chains during the acid solubilization of the microcrystalline structure determine the interactions with the Ti precursor during impregnation and the transformations of both cellulose and Ti phases during thermal treatments. Thus, the chemical nature and physicochemical properties of the active phases can be fitted by adjusting the surface chemistry and carbonization temperature. 

Phosphorus functionalities, phosphate groups in particular, avoid crystal growth when composites are carbonized at low temperature (500 °C), while oxygen functionalities favor the transformation of anatase nanocrystals into rutile phase. With increased carbonization temperature (800 °C), Ti precursors react with the phosphate surface groups, forming TiP_2_O_7_ nanocrystals; sintering is also favored, and Ti^+4^ is partially reduced to Ti^+3^. These interactions with the carbon phase produce a significant narrowing of the band gap regarding rutile or TiP_2_O_7_ pure phases. The combination of a developed porosity and surface area, a narrow band gap and small crystal size favor the dye adsorption and prevent the electron–hole recombination, consequently enhancing the dye mineralization in all carbon–Ti composites compared to that of the commercial P25.

## Figures and Tables

**Table 1:** Surface composition of Ti-impregnated cellulose and its carbon derivatives determined by X-ray photoelectron spectroscopy (XPS) and measurements of pH_PZC_.

Sample	pH_PZC_ (±0.2)	Atomic Content (±0.1 wt. %)				
		C	O	N	P	Ti
CPTi	n.d.	8.5	47.3	-	26.8	17.4
CPTi1	2.7	22.0	42.7	-	21.9	13.4
CPTi2	2.8	27.3	36.4	-	22.8	13.5
CNTi	n.d.	18.7	36.2	1.2	-	43.9
CNTi2	6.5	16.4	35.4	-	-	48.2

n.d. = not determined.

**Table 2:** Textural characteristics of the carbon–Ti composites.

Sample	*S_BET_* (±5 m^2^·g^−1^)	*S_micro_* (±5 m^2^·g^−1^)	*L_0_* (±0.1 nm)	*W_0_* (±0.01 cm^3^·g^−1^)	*V_meso_* (±0.01 cm^3^·g^−1^)
CP1	17	20	2.2	0.01	0.02
CP2	552	624	1.5	0.22	0.07
CN1	226	253	1.5	0.09	0.06
CN2	115	122	1.7	0.04	0.06
CPTi1	28	35	1.4	0.01	0.27
CPTi2	30	48	1.8	0.02	0.35
CNTi1	124	141	1.6	0.05	0.13
CNTi2	319	360	1.5	0.13	0.34

**Table 3:** Removal of OG obtained by photocatalysis using different TiO_2_-based materials.

Material	OG Concentration	Irradiation Source	Removal (%)	Reference
TiO_2_ (Degussa P25)	84.2 μM, 2.5 g/L catalyst	UV high-pressure mercury lamp	100%, 120 min	[56]
Sn(IV)/TiO_2_/AC	110.5 μM, 12.5 g/L catalyst	UV high-pressure mercury light	99.1%, 60 min	[57]
TiO_2_ (99% anatase) on glass plates	66.3 μM	UV lamp (λ = 365 nm)	100%, 130 min	[58]
Au–TiO_2_	25 μM	UV low-pressure mercury lamp	100%, 60 min	[59]
10%CNT–TiO_2_	110.5 μM, 1 g/L catalyst	Metal halide lamp + cut-off filter (λ > 400 nm)	100%, 120 min	[60]
CX–TiO_2_	55.3 μM, 1 g/L catalyst	Vis TeptoLux 2.0 lamp	90%, 400 min	[4]
g-C_3_N_4_–TiO_2_	200 μM, 0.5 g/L	Simulated solar light (Xe Lamp)	82%, 10 min	[61]
rGO–TiO_2_	10 μM	Microwave irradiation	88%, 20 min	[62]
CNTi1	22 μM, 1.0 g/L	UV low-pressure mercury lamp	99%, 40 min	This work
CPTi1	22 μM, 1.0 g/L	UV low-pressure mercury lamp	100%, 40 min	This work
TiO_2_ (Degussa P25)	22 μM, 1.0 g/L	UV low-pressure mercury lamp	79%, 40 min	This work

AC = activated carbon; CNT = carbon nanotubes; CX = carbon xerogel; rGO = reduced graphene oxide.

## Supplementary Materials

The following are available online at https://www.mdpi.com/2079-4991/10/4/729/s1: Figure S1: High-resolution XPS spectra of the P2p region for Ti-impregnated cellulose derivatives and their corresponding carbonized composites.
