# Supplementary material for: Functionalized Cellulose for the Controlled Synthesis of Novel Carbon–Ti Nanocomposites: Physicochemical and Photocatalytic Properties
Source: Nanomaterials (Basel). 2020 Apr 11;10(4):729. doi: 10.3390/nano10040729 (PMC7221653; doi:10.3390/nano10040729)
Supplement: Supplementary file 1 [file nanomaterials-10-00729-s001.pdf]

nanomaterials

Supplementary materials

Functionalized Cellulose for the Controlled Synthesis of Novel Carbon-Ti Nanocomposites: Physicochemical and Photocatalytic Properties

Hesham Hamad [1,2], Esther Bailón-García [1], Sergio Morales-Torres [1,*], Francisco Carrasco-Marín [1], Agustín F. Pérez-Cadenas [1] and Francisco J. Maldonado-Hódar [1]

1 Carbon Materials Research Group, Department of Inorganic Chemistry. Faculty of Sciences. University of Granada. Avda. Fuente Nueva, s/n. ES18071 Granada, Spain; heshamaterials@hotmail.com (H.H.); ebailon@ugr.es (E.B.-G.); fmarin@ugr.es (F.C.-M.); afperez@ugr.es (A.F.P.-C.); fjmaldon@ugr.es (F.J.M-H).

2 Permanet address: Fabrication Technology Department, Advanced Technology and New Materials Research Institute (ATNMRI), City of Scientific Research and Technology Applications (SRTA-City), New Borg El-Arab City 21934, Egypt

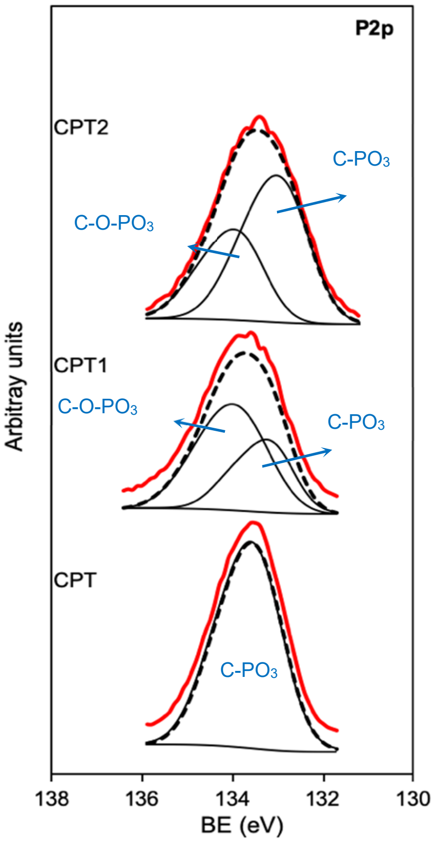

Figure S1. High resolution XPS spectra of the P2p region for Ti-impregnated cellulose derivatives and their corresponding carbonized composites.
